# Ruxolitinib and exemestane for estrogen receptor positive, aromatase inhibitor resistant advanced breast cancer

**DOI:** 10.1038/s41523-022-00487-x

**Published:** 2022-11-11

**Authors:** Igor Makhlin, Nicholas P. McAndrew, E. Paul Wileyto, Amy S. Clark, Robin Holmes, Lisa N. Bottalico, Clementina Mesaros, Ian A. Blair, Grace R. Jeschke, Kevin R. Fox, Susan M. Domchek, Jennifer M. Matro, Angela R. Bradbury, Michael D. Feldman, Elizabeth O. Hexner, Jacqueline F. Bromberg, Angela DeMichele

**Affiliations:** 1 Division of Hematology/Oncology, University of Pennsylvania, Perelman School of Medicine, Philadelphia, PA USA; 2 Division of Hematology/Oncology, UCLA David Geffen School of Medicine, Los Angeles, CA USA; 3 Center for Clinical Epidemiology and Biostatistics, University of Pennsylvania, Philadelphia, PA USA; 4 University of Pennsylvania, Abramson Cancer Center, Philadelphia, PA USA; 5 Department of Systems Pharmacology and Translational Therapeutics, University of Pennsylvania, Philadelphia, PA USA; 6 Center for Excellence in Environmental Toxicology, Department of Systems Pharmacology and Translational Therapeutics, University of Pennsylvania, Philadelphia, PA USA; 7 Georgetown University, Washington, DC USA; 8 Basser Center at the University of Pennsylvania, Philadelphia, PA USA; 9 Division of Hematology/Oncology, UC San Diego, San Diego, CA USA; 10 Department of Pathology, Perelman School of Medicine, University of Pennsylvania, Philadelphia, PA USA; 11 Memorial Sloan Kettering Cancer Center, New York, NY USA

**Keywords:** Predictive markers, Breast cancer, Tumour biomarkers, Translational research

## Abstract

Circulating IL-6, an activator of JAK/STAT signaling, is associated with poor prognosis and aromatase inhibitor (AI) resistance in hormone-receptor positive (HR+) breast cancer. Here we report the results of a phase 2 single-arm Simon 2-stage trial combining Ruxolitinib, an oral selective inhibitor of JAK1/2, with exemestane, a steroidal AI, in patients with HR+ metastatic breast cancer (MBC) after progression on non-steroidal AI (NSAI). Safety and efficacy were primary objectives, and analysis of inflammatory markers as predictors of response was a key secondary objective. Twenty-five subjects enrolled. The combination of ruxolitinib and exemestane was safe, though anemia requiring transfusion in 5/15 (33%) at the 25 mg dose in stage 1 led to a reduction to 15 mg twice daily in stage 2 (with no additional transfusions). Clinical benefit rate (CBR) in the overall study population was 24% (95% CI 9.4–45.1); 6/25 patients demonstrated stable disease for ≥6 months. Median progression-free survival was 2.8 months (95% CI 2.6–3.9). Exploratory biomarkers revealed high levels of systemic inflammation and 60% harbored a high-risk IL-6 genotype. Pharmacodynamics demonstrated modest on-target inhibition of phosphorylated-STAT3 by ruxolitinib at a tolerable dose. Thus, ruxolitinib combined with exemestane at a tolerable dose was safe but minimally active in AI-resistant tumors of patients with high levels of systemic inflammation. These findings highlight the need for more potent and specific therapies targeting inflammation in MBC.

## Introduction

Despite the use of adjuvant endocrine therapy with tamoxifen and aromatase inhibitors (AIs), 20% of patients with newly diagnosed early-stage estrogen receptor-positive (ER+) breast cancer (BC) will relapse within 10 years of diagnosis and additional relapse risk continues for the duration of a patient’s lifetime^1^. While an endocrine therapy-containing regimen with a CDK4/6 inhibitor is typically the optimal first line therapy in metastatic ER+ BC, establishing drug regimens that address eventual resistance remains an unmet clinical need^2^. The mechanisms underlying resistance to AIs have been a major area of research, with inflammatory pathways implicated as one possible mechanism.

In vitro studies have linked interleukin-6 (IL-6) to poor prognosis in BC via activated Janus kinase (JAK)/STAT tumor signaling^3,4^, leading to an aggressive phenotype. Elevated serum levels of C-reactive protein (CRP) and serum amyloid A (SAA), both of which are downstream products of IL-6, have also been associated with reduced disease-free survival (DFS) as well as worse overall survival (OS) in patients with early-stage BC^5–7^. Moreover, polymorphisms in the IL-6 promoter that functionally increase IL-6 transcription have been shown to be associated with poor prognosis and early relapse in women with ER+, node-positive BC^8^. This may be partially explained by the upregulation of aromatase that is seen with elevated levels of IL-6^9^, as well as the relative upregulation of the soluble IL-6 receptor in ER+ BC as compared to triple-negative BC^10^. IL-6 is also implicated as a driver of resistance to endocrine therapy (ET) in ER+ metastatic BC (MBC). Preclinical work has shown that IL-6/STAT3 signaling takes control of a subset of shared IL-6/ER-enhancers to both drive BC invasion and render the BC resistant to ET, and that this process can be inhibited through blockade of IL-6/STAT3 with ruxolitinib, an oral JAK1/2 inhibitor^11^. ET has also been shown to increase paracrine levels of IL-6 and enrich for a metabolically-dormant, ET-resistant CD133^high^/ER^low^/IL-6^high^ cancer stem-cell population; blockade of IL-6 receptor restores ET-sensitivity via re-expression of the estrogen receptor, suggesting IL-6 as a driver of ET-resistance and therefore an attractive target in patients with ER+ MBC that is resistant to AI^12^.

Despite strong pre-clinical rationale for IL-6/STAT3 blockade, early phase studies utilizing ruxolitinib (INCB018424), an orally available selective JAK1/2 inhibitor currently approved by the Food and Drug Administration (FDA) for use in myelofibrosis, polycythemia vera, and graft-versus-host disease^13^ have failed to show clinically meaningful responses in metastatic breast cancer as a single agent^14^ or in combination with chemotherapy^15,16^ or trastuzumab^17^. However, no studies to date have examined its feasibility and activity in combination with endocrine therapy in the ET-resistant metastatic setting. Exemestane, an irreversible steroidal AI that is structurally related to the natural substrate androstenedione^18–21^ is approved in adjuvant and metastatic ER+ BC, and has demonstrated an objective response rate (ORR) of 6.7% and a clinical benefit rate (CBR) of 31.5% as a single agent in ER+ MBC progressing on previous non-steroidal AI^22^. The objective of the “JAKEE” Trial (NCT01594216) is to determine whether addition of ruxolitinib to exemestane is safe and could restore ET-sensitivity in women with ER+ BC who had relapsed or progressed after NSAI therapy, and to determine if biomarkers reflecting enhanced IL-6 signaling in the tumor microenvironment, such as increased serum inflammatory markers, increased estradiol levels and/or germline polymorphisms in host IL-6 promoter could identify participants more likely to respond to the combination.

Herein we show that ruxolitinib added to exemestane, although safe and tolerable at a dose of 15 mg twice daily, does not meet pre-specified response criteria to merit further investigation. Through pharmacodynamic studies, we show that ruxolitinib has only a modest on-target inhibition of phospho-STAT3, potentially explaining the efficacy results. Finally, analysis of inflammatory markers demonstrates high levels of systemic inflammation and a high frequency of high-risk IL-6 promoter polymorphisms in this population though these markers do not discriminate between responders and non-responders.

## Results

### Study Population

A total of twenty-five subjects were enrolled from 10/22/12 to 1/14/16. Fifteen enrolled in the first stage and 10 in the second stage. The demographic and clinical features of these subjects are shown in Table 1. Notably, 92% identified as Caucasian and only 4% identified as African American. All patients had progressed on prior endocrine therapy in the metastatic setting. Approximately one-third of patients had bone-only disease and 20% had known, clinically stable CNS disease. Two participants (8%) had HER2+ disease on prior biopsies. Importantly, 28% had already received ≥2 lines of chemotherapy in the metastatic setting.

**Table 1:** Study Population.

Clinical Characteristics	Value
Total Enrolled	25 (100%)
Age (Median, range)	59 (34–85)
Race	White (92%), Black (4%), Unknown (4%)
BMI (Median, range)	27.5 (18.1–55.1)
Sites of disease	
Bone only	32%
Visceral only	20%
Visceral and bone	44%
CNS	20%
Prior Lines of Metastatic Chemotherapy	0
0	44%
1	28%
2 or more	28%
Prior Endocrine Therapy	
Adjuvant	16 (64%)
Metastatic	25 (100%)

### Safety

Table 2 describes the major toxicities for the combination of ruxolitinib and exemestane. The most common grade ≥2 toxicities were anemia, hypertension, fatigue, and leukopenia. The majority of AE’s were grade 1 or 2. There were no grade 5 events.

**Table 2:** Most Common Adverse Events (CTCAE G ≥ 2).

Toxicity	Grade 2, *n* (%)	Grade 3, *n* (%)	Grade 4, *n* (%)	Total (%)
Anemia	5 (20%)	4 (16%)	0	36%
Hypertension	4 (16%)	4 (16%)	0	32%
Fatigue	4 (16%)	4 (16%)	0	32%
Leukopenia	4 (16%)	3 (12%)	0	28%
Depression	2 (8%)	3 (12%)	0	20%
Anxiety	5 (20%)	0	0	20%
ANC, decreased	2 (8%)	3 (12%)	0	20%
Insomnia	3 (12%)	0	0	12%
Hypothyroidism	3 (12%)	0	0	12%
Nausea	1 (4%)	1 (4%)	0	8%
Anorexia	2 (8%)	0	0	8%
Dizziness	2 (8%)	0	0	8%
Dyspnea	2 (8%)	0	0	8%
Cough	2 (8%)	0	0	8%
Urinary Tract Infection	2 (8%)	0	0	8%
Thrombocytopenia	2 (8%)	0	0	8%

Of the 15 subjects enrolled into the first stage, seven (46.7%) required dose reductions for ruxolitinib, primarily due to anemia, a known on-target (JAK2) toxicity, including 3 (20%) that required two dose reductions (20 mg followed by 15 mg). Five out of 15 subjects (33.3%) required PRBC transfusions, though none of the patients discontinued treatment. Thus, while not formally crossing the safety stopping boundary, the severity of the anemia led to a decision to decrease the dose of ruxolitinib to 15 mg twice daily for the second stage to optimize patient safety and tolerability. Ten additional subjects enrolled onto the second stage; 1 subject required a dose reduction and no patients required RBC transfusion. Only 1/25 subjects discontinued the trial due to toxicity, hence the primary objective of safety was met.

### Efficacy

Of the 25 participants enrolled, 21 were evaluable for assessment of PR or CR by RECIST 1.1 criteria, as 4 had bone-only disease that was only evaluable for SD. None achieved a complete or partial response. Six participants (24%) achieved stable disease for ≥6 months, for a CBR of 24% (95% CI 9.4–45.1). Notably, the majority of participants in this trial had fairly rapid progression: 13 (52%) had evidence of progression at the first imaging time point (3 months), and an additional 6 (24%) had progression before the first imaging time point. Supplementary Fig. 1 shows the number of cycles completed per participant on trial. Participants completed a median of 3 cycles on trial (range 1–21). The median PFS for this cohort was 2.8 months (95% CI 2.6–3.9), as seen on the Kaplan Meier Plot in Supplementary Fig. 2.

#### Pharmacodynamic assessment of ruxolitinib effect on JAK/STAT signaling

**Fig. 1:**
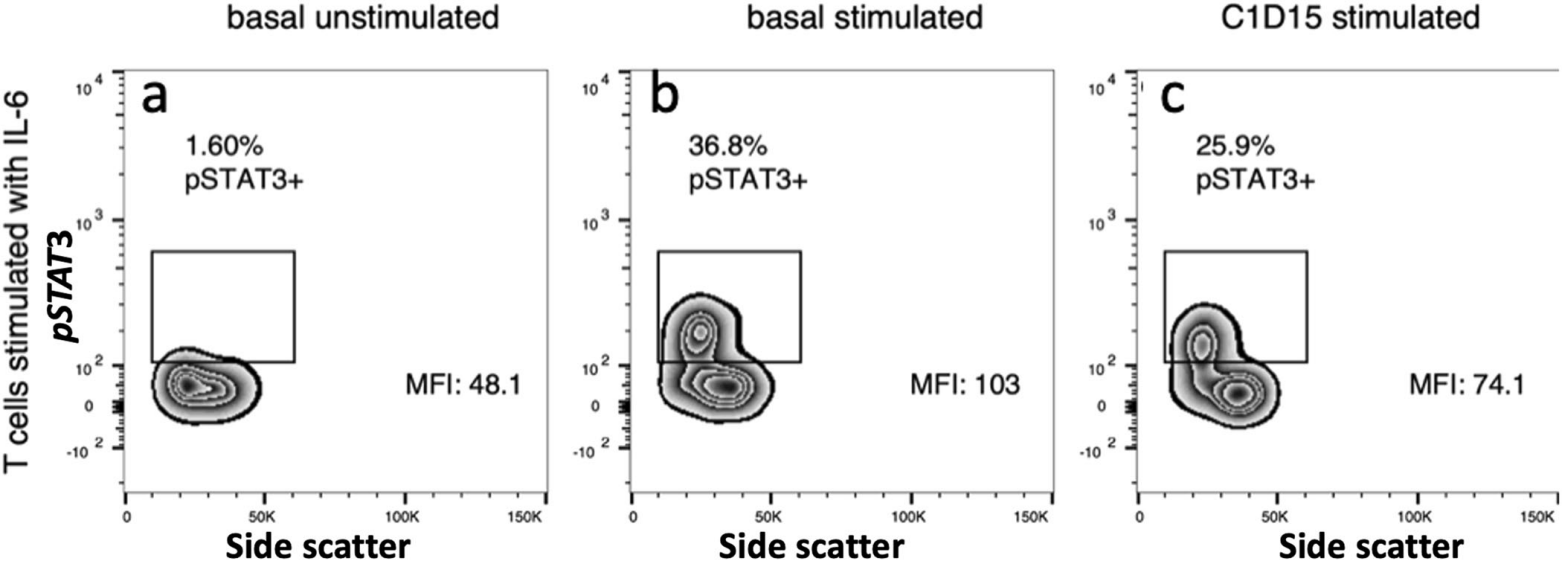
Pharmacodynamic Analysis of Ruxolitinib Target Inhibition. Representative contour plots for a participant on trial who progressed at 3 months. Peripheral blood was collected from participants at baseline and on-treatment (cycle 1 day 15). CD3+ T cells were assessed for baseline phosphoSTAT3 (pSTAT3) activity using flow cytometry. Exogenous cytokine stimulation with IL-6 was performed at baseline and at cycle 1 day 15 samples to assess extent of inhibition by Ruxolitinib. CD3+ gated cells: **a**: at baseline without cytokine stimulation **b**: at baseline with IL-6 **c:** day 15 with IL-6.

Seventeen patients had a baseline sample and at least one on-treatment trough sample for analysis. Without cytokine stimulation, there was no detectable basal phosphoSTAT3/STAT5, as expected based on prior studies in hematologic malignancies^23^. A representative contour plot for pSTAT3 is shown in Fig. 1a. In the presence of cytokine stimulation, phosphorylation increased in all untreated samples (Fig. 1b). In 16 of 17 participants, the T cell response to IL-6, as measured by phosphoSTAT3, was blunted in CD3+ cells in trough samples from treated patients (Fig. 1c). The median inhibition for all 17 participants was 25% (range 0–77%). Similar results were seen for pSTAT5 in T cells stimulated with IL-6 (not shown) and in granulocytes stimulated with G-CSF (Supplementary Fig. 3). There was no differential effect of percent inhibition of phosphoSTAT3 in responders (*n* = 5) vs non-responders (*n* = 12) (median inhibition 20% vs 29%, chi-squared *p* = 0.15), and there was no difference in median % inhibition by the trial stage, corresponding to 25 mg twice daily vs 15 mg twice daily (24.5% vs 29%, chi squared *p* = 0.49).

#### Serum Inflammatory Markers

Nineteen participants (76%) had available data regarding inflammatory marker levels at C1D1, C1D15, and C2D1. Mean baseline levels of CRP, SAA, and IL-6 are listed in Table 3 and were found to be elevated beyond normal upper limits as defined by the laboratory of the Hospital of the University of Pennsylvania (provided in Table 3). Distributions for the inflammatory markers are illustrated via histograms in Supplementary Fig. 4 (Panels a, c, e).

**Table 3:** Distribution of IL-6 genotypes and baseline inflammatory & estrogen levels.

Inflammatory Biomarker	Value
Inflammatory Serum Biomarkers [Upper limit of normal], Mean (Range)	
CRP [8 mg/L]	33.5 (0.2–146.8)
SAA [10 mg/L]	26.1 (2.8–162.5)
IL-6 [2 pg/mL]	4.9 (1.8–11.5)
IL-6 Genotype (Frequency)	
−174	C/C 16%
	G/C 24%
	G/G 60%
−572	G/C 16%
	G/G 84%
−597	A/A 16%
	G/A 24%
	G/G 60%
Estrogen Metabolites, Mean (Range)	
Estrone (E1, pg/mL)	157.0 (0.2–1039.0)
Estradiol (E2, pg/mL)	7.1 (0.2–44.1)

**Fig. 2:**
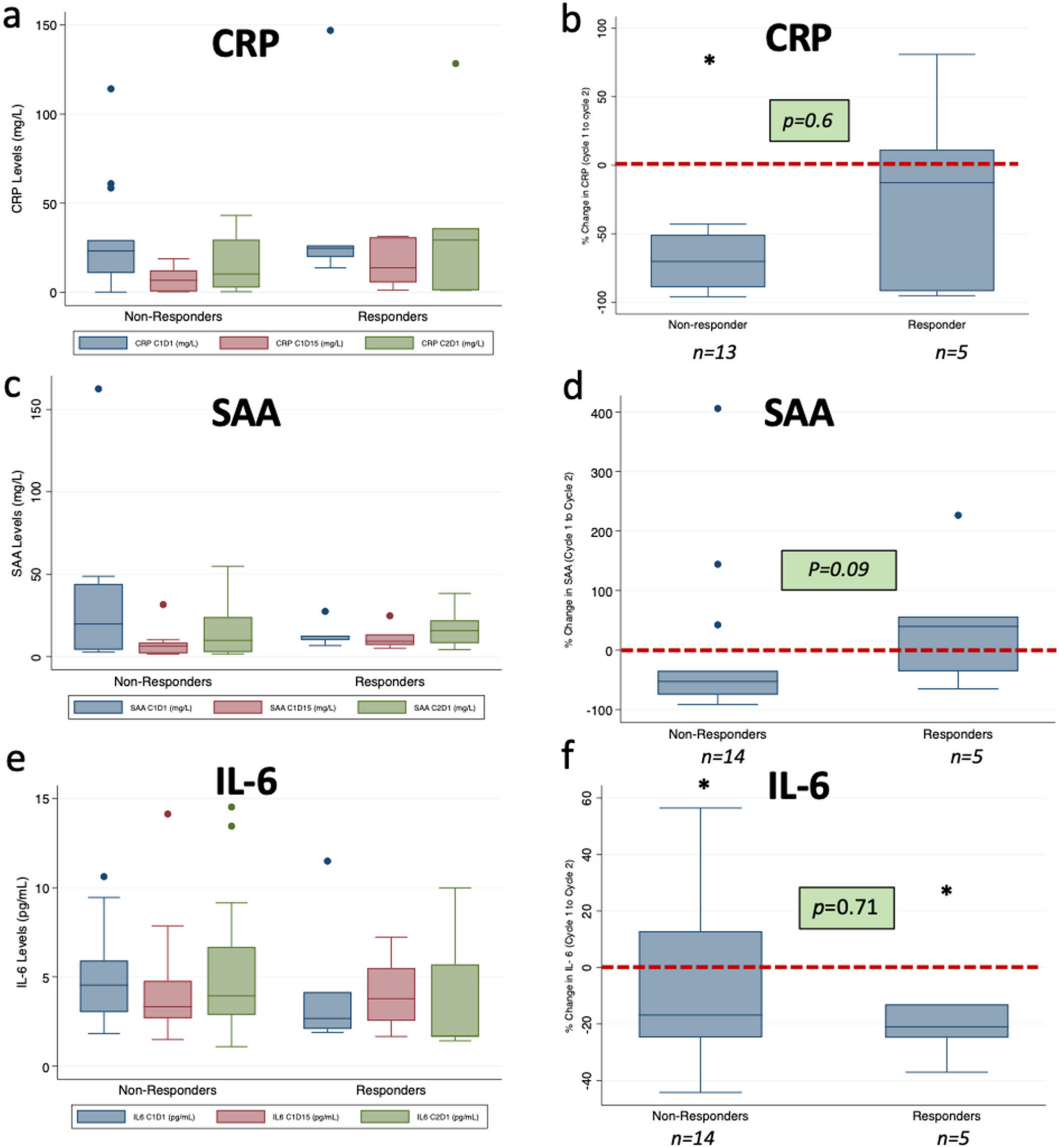
Change in inflammatory markers by response group. Box plots representing level of inflammatory markers over time (**a**, **c**, **e**) and % change from pre-treatment to cycle 2 day 1 (**b**, **d**, **f**). There were no significant differences in changes from baseline to cycle 2 day 1 between responders and non-responders. Whisker endpoints represent range (minimum-maximum), box limits represent interquartile range (upper and lower quartiles), and center line represents the median. Points above the box-and-whiskers represent outliers. Horizontal dashed red line in panels **b**, **d**, **f** represent zero line for reference.

There was no difference in baseline levels of CRP and IL-6 between responders (*n* = 5) and nonresponders (*n* = 14) to study therapy, although there was a non-significant trend towards lower baseline SAA levels among responders (Supp. Fig. 4, panels b, d, f). For most participants, there appeared to be an initial drop in levels of CRP, SAA, and IL-6 by cycle 1 day 15, with rebound in levels by cycle 2 day 1, although the percentage change in levels from baseline to cycle 2 day 1 was not significantly different between responders and non-responders (Fig. 2).

#### IL-6 promoter polymorphisms

The frequencies of each genotype in this cohort are listed in Table 3. In this study population, there was a 1:1 correlation between −174 G/G and −597 G/G. Fifteen of 25 (60%) were classified as having a high-risk IL-6 polymorphism. There was no significant difference in the frequency of high-risk IL-6 promoter polymorphisms between responders and non-responders (50% vs 63%, chi-squared *p* = 0.65). Of note, levels of baseline and cycle 2 day 1 circulating inflammatory markers did not significantly differ by IL-6 promoter status, though there was a trend towards greater SAA at cycle 2 day 1 in those that had high-risk IL-6 promoter (Supplementary Fig. 5).

#### Estrogen metabolites

**Fig. 3:**
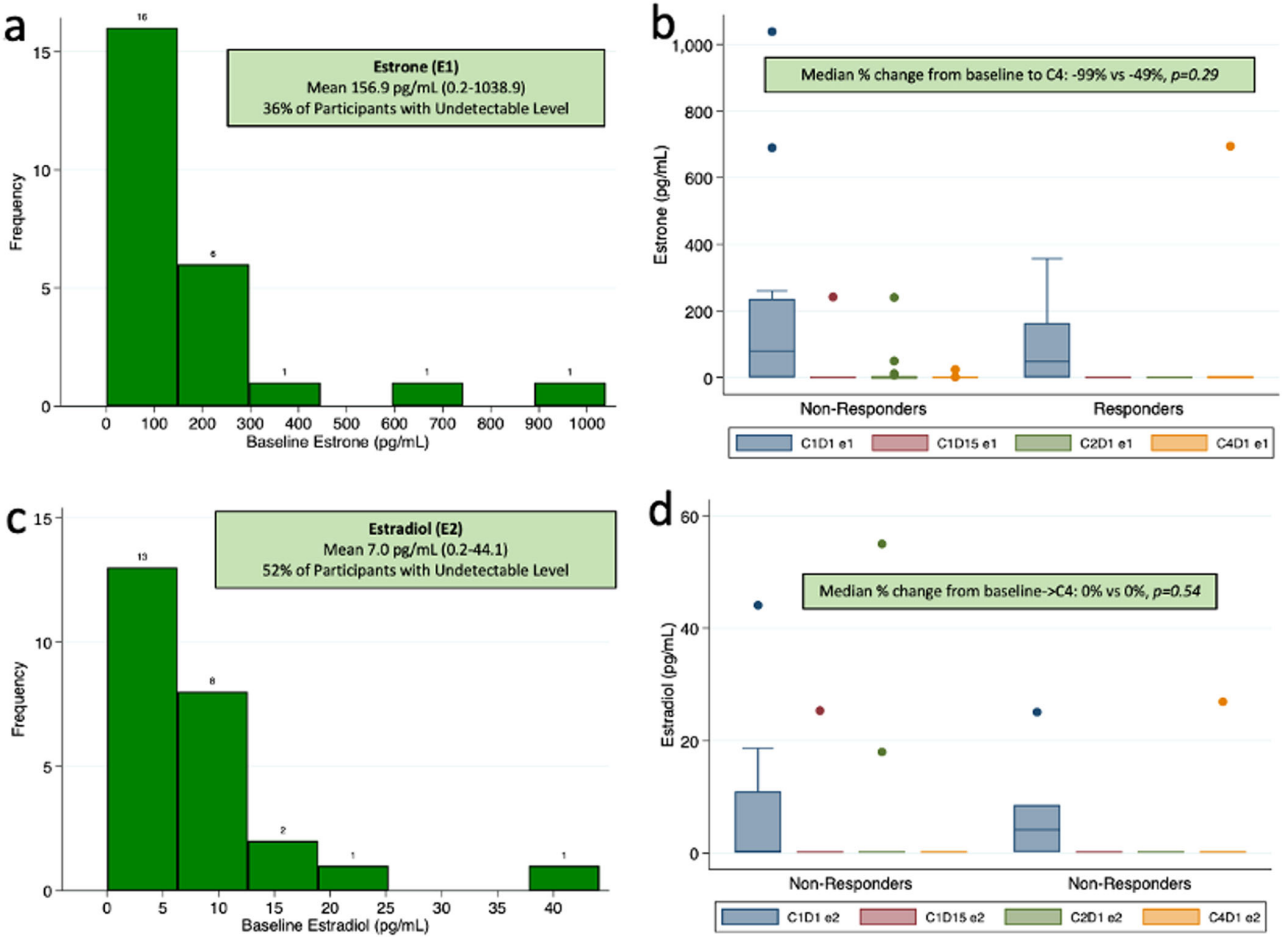
Baseline and change in estrogen levels by response group. Left column shows histogram distributions of baseline estrone (panel **a**) and estradiol (panel **c**) levels for the full cohort. Numbers above each bar represent frequency (corresponding to vertical axis). Right column shows box plots for levels of E1 (panel **b**) and E2 (panel **d**) over 4 time points: pre-treatment (cycle 1 day 1), cycle 1 day 15, cycle 2 day 1, and cycle 4 day 1. Non-parametric testing was employed to test for difference between the percent change in E1 and E2 from baseline to cycle 4 day 1, with *p* value representing equality of median test. Whisker endpoints represent range (minimum-maximum), box limits represent interquartile range (upper and lower quartiles), and center line represents the median. Points above the whiskers and boxes represent outliers.

All 25 subjects had estrone (E1) and estradiol (E2) levels analyzed at baseline and cycle 1 day 15, cycle 2 day 1, and cycle 4 day 1. Baseline levels for the cohort are listed in Table 3. Figure 3 (panels a, c) shows histograms representing the baseline distribution for E1 and E2 for the full cohort. 36% and 52% of subjects had levels of E1 and E2 below the level of detection, respectively. Notably, there was no difference in the proportion of participants above the median baseline levels or percent undetectable E1 and E2 between responders and non-responders (% undetectable at baseline, E1: 36.9% vs 33.3%, *p* = 1.0, E2: 52.6% vs 50%, chi-squared *p* = 1.0). There was also no difference in the percent change in E1 or E2 levels from baseline to cycle 4 day 1 by responder status (Fig. 3, panels b, d).

As IL-6 has been shown to upregulate aromatase activity and thus lead to increased levels of estrogens, we investigated the association between IL-6 genotypes and E1/E2 levels. Only 20% of subjects with a high-risk IL-6 promoter polymorphism had undetectable baseline E1 levels, compared with 60% of those without the high-risk promoter (chi-squared *p* = 0.041), and lack of suppression of E1/E2 on treatment was restricted to those with high-risk IL-6 genotypes (Supplementary Fig. 6). There was no significant difference for baseline E2 levels by high-risk IL-6 status (60% vs 46.6%, chi-squared *p* = 0.51). Given the influence of body mass index (BMI) on estrogen levels, the relationship among estrogen levels, IL-6 promoter status, and BMI was assessed. There was no statistically significant difference in the frequency of undetectable baseline estrogens between obese and nonobese participants (E1: 28.6% vs 71.4%, *p* = 0.63; E2 28.6% vs 71.4%, chi-squared *p* = 0.14). Univariate logistic regression identified a significant relationship between odds for undetectable baseline E1 and presence of high-risk IL-6 promoter (OR 0.17, *p* = 0.05) but not by obesity (OR 0.97, *p* = 0.50). No significant associations were found for baseline E2 by either IL-6 promoter genotype or obesity. By univariate logistic regression analysis, there was no association between baseline levels of E1 or E2 and response rate (E1 OR 0.99 [95% CI 0.99–1.00], *p* = 0.54); E2 OR 1.00 [95% CI 0.91–1.10], *p* = 0.99).

## Discussion

In this phase II single-arm trial, the combination of ruxolitinib and exemestane administered to women with metastatic ER+ BC having relapsed or progressed on an NSAI was safe and feasible. However, the ability to fully dose ruxolitinib was limited by excessive anemia requiring transfusion in one-third of the patients in the first stage, leading to a reduction in ruxolitinib from the 25 mg approved dose to 15 mg in the second stage. The overall response to this combination was minimal with no CR or PR and stable disease in 24%. The median PFS for the study population overall was only 2.8 months (95% CI 2.6–3.9). Our pharmacodynamic and correlative biomarker analyses suggest that the lack of meaningful clinical responses observed are likely due to multiple factors including a sub-optimal on-target pharmacologic effect at the tolerable dose and a study population with advanced, treatment-resistant clinical features and highly inflamed tumors.

Only 6 participants (24%) achieved stable disease for ≥6 cycles, with the longest responder demonstrating disease control for approximately 21 cycles. To put this in context of other published studies utilizing exemestane in a similar setting, the CBR of participants treated with single-agent exemestane in the EFFECT trial (comprising those with ER+ MBC progressing on NSAI) was 31.5%^22^; however, the JAKEE study population was considerably more advanced, with 20% of the study population having brain metastases (EFFECT excluded brain metastases) and a higher proportion of prior chemotherapy use in the metastatic setting (28% EFFECT vs 89% JAKEE). Therefore, the clinical features of the JAKEE population likely represent a more treatment-resistant, aggressive phenotype and thus contributed to a lower response rate in our cohort. Additionally, it’s likely that some proportion of patients in this cohort harbored an ESR1 mutation - a known resistance mechanism of breast cancer to AI’s with a prevalence reported as high as 53% or greater in MBC^24^, though this was not assayed in this trial.

In addition to the high-risk clinical features, we hypothesized that systemic inflammation through IL-6/JAK/STAT signaling renders tumors more treatment-resistant due to multiple factors, including IL-6 effect on estrogen levels and JAK/STAT pro-survival signaling (and therefore that targeting the JAK/STAT pathway with ruxolitinib may augment response with exemestane). Through analysis of circulating inflammatory markers and IL-6 promoter genotyping, our cohort was notable for highly inflammatory features, including higher levels of baseline circulating CRP than previously published cohorts of metastatic BC patients^25^ and a 60% frequency of high-risk IL-6 genotypes. While there are no data detailing IL-6 promoter genotype frequencies in the metastatic setting, studies in the early-stage setting demonstrate a range of frequencies for high-risk IL-6 promoter polymorphisms, but consistently show an association for elevated risk for relapse. In the Wellness After Breast Cancer-II (WABC-II) cohort, which comprised post-menopausal women with stage I-III BC (ER+ and/or HER2+), the presence of a high-risk IL-6 promoter genotype (−174G/G or −597G/G, frequency 58%) conferred an increased risk for breast cancer relapse among the ER+ subjects (odds ratio 2.35 [95% CI 1.16–4.77, *p* = 0.018])^7^. Interestingly, the HER2+ cohort had an even higher frequency of high-risk genotypes among cases that relapsed compared with controls (82% vs 45%, *p* = 0.024), with an odds ratio for relapse of 7.19, 95% CI 1.47–35.30, *p* = 0.015), suggesting a possible link between HER2-signaling and inflammation^7^; this hypothesis is supported by recent preclinical in vitro/in vivo models by Hartman et al, who showed that HER2-mediated transformation/tumorigenesis is dependent on IL-6 secretion^26^. In the ECOG 2190 trial which comprised patients with very high risk (≥10 positive lymph nodes) locally advanced BC, the presence of a high-risk IL-6 genotype conferred an increased risk of a DFS event in the ER+ subset (−174G/G, frequency 38%, HR 1.71 [1.16–2.52], −597G/G, frequency 39%, HR 1.60 [1.09–2.35])^8^. Interestingly, no effect was seen among the ER- subjects. Taken together with our metastatic trial, these data suggest that patients enriched for an inflammatory state as measured by IL-6 promoter genotyping represent a more treatment-resistant population. Unlike circulating inflammatory proteins (e.g. CRP, IL-6), which exhibit diurnal variation and thus introduce potential for measurement error, IL-6 promoter genotyping represents a stable ‘fingerprint’ of systemic inflammation, which may explain why the WABC-II study detected an association between IL-6 promoter genotype and relapse, but not with circulating IL-6 cytokine levels.

This link between high-risk IL-6 promoter genotypes and treatment resistance can at least partly be explained by IL-6’s known action of inducing the aromatase enzyme, particularly in the breast cancer microenvironment, increasing local levels of estrogens in an autocrine fashion and thereby promoting growth and survival^27,28^. Importantly, we found that those participants with high-risk IL-6 genotypes had higher baseline levels of E1, and that virtually all cases where therapy was unable to suppress estrone/estradiol levels while on treatment were restricted to those with high-risk IL-6 genotypes. Notably, BMI, a potential effect modifier, did not appear to modify or confound these findings.

Given that our cohort exhibited inflammatory features that we hypothesized should be amenable to modulation with ruxolitinib, we utilized a flow cytometry-based pharmacodynamic assay using peripheral blood samples (validated in hematologic malignancies as a non-invasive surrogate for on-target effect^23^) and showed that ruxolitinib administered at 15 mg or 25 mg twice daily achieved only a modest inhibition of phospho-STAT3 with a median inhibition of 25% (with similar results for phospho-STAT5); this may explain the lack of suppression of estrogen levels in those with high-risk IL-6 genotypes on treatment with ruxolitinib and exemestane. Although few studies have published pharmacodynamic investigations of ruxolitinib in MBC, our results compare similarly with those of Stover et al., who reported on a limited subgroup of 3 subjects with metastatic triple-negative breast cancer that had received single-agent ruxolitinib and had paired pre-treatment and on-treatment tumor biopsies; they demonstrated incomplete suppression, with only a 40–55% decrease in phostpho-STAT3 with treatment^14^. Of note, there were no objective responses in that clinical trial comprising 21 subjects. These data suggest that the currently approved doses of ruxolitinib may be insufficient to achieve the potent inhibition of the IL-6/JAK/STAT pathway necessary to induce clinically meaningful responses, and that either novel drug-to-target delivery mechanisms are needed to bypass systemic toxicity (thus allowing higher doses to be given safely), or more specific JAK inhibitors need to be tested to minimize off-target toxicity while maximizing on-target suppression. Our results add to the growing body of evidence that utilizing ruxolitinib in different MBC settings (ER+^15^, HER2+^17^, and triple negative^14,16^) is not effective, which may be explained in part by sub-optimal suppression of the JAK/STAT pathway.

We powered our study to detect a response rate of 25%, but it’s possible that the true effect size was smaller than our sample size would have been able to detect. As the study enrolled from 2012 to 2016, only 2 subjects (8%) had received prior CDK4/6 inhibitors (both on clinical trials), thus this cohort does not necessarily represent the modern approach of first-line CDK4/6 inhibitor combined with ET; however, given the negative results of this study, it’s unlikely that a higher proportion of subjects with prior CDK4/6 inhibitor exposure would have led to higher response rates; rather, it’s more likely the cohort would have had a lower clinical benefit rate. Measurement of inflammatory cytokines can be imprecise given their significant diurnal variation^29^ and influence from other competing factors (inflammation, stress, other medicines, etc.); thus, there is potential for measurement error since the timing blood draws was not consistent, compounded by the small sample size and resultant larger variability. However, we also assayed for host IL-6 promoter polymorphisms, which unlike circulating markers, are constant, not subject to external signals such as stress and time of day, and have been shown to correlate with higher circulating inflammatory marker levels^30^. Finally, although we were able to confirm only modest inhibition of pSTAT3 with cycle 1 day 15 samples, the lack of on-treatment tumor biopsies or later time point blood draws precluded our ability to confirm durable suppression or examine the extent of tumor heterogeneity in pSTAT3 activation; it’s possible that the effect of pSTAT3 suppression was transient, which could potentially explain the rebound effect seen by cycle 2 day 1 in most participants’ inflammatory markers.

In summary, the combination of exemestane and ruxolitinib was feasible, with treatment-emergent anemia ameliorated after a dose reduction of ruxolitinib to 15 mg twice daily; however, this combination did not meet pre-specified response criteria to merit further investigation. Biomarker studies revealed a high proportion of patients with high-risk IL-6 promoter polymorphisms, though ruxolitinib achieved only a modest inhibition of the IL-6/JAK/STAT pathway. Thus, novel approaches for potent inhibition of JAK/STAT signaling with a tolerable safety profile as well as other inhibitors of inflammatory pathways are needed.

## Methods

### Eligibility and enrollment

Eligible participants had histologically confirmed metastatic ER+ BC (defined as ≥5% by immunohistochemistry performed by clinical pathologists in a CLIA laboratory) on either a primary or metastatic tumor biopsy and were post-menopausal (either surgically via oophorectomy or no menses in the previous 12-month period). Those that were also HER2+ by IHC (3+) or FISH (by ASCO/CAP guidelines) were eligible to participate. Patients who were premenopausal at diagnosis and rendered amenorrheic by tamoxifen were required to have a serum estradiol level <30 pg/ml after discontinuation of tamoxifen. Participants must have either relapsed within 2 years of completing adjuvant NSAI or progressed on one in the metastatic setting. Bone-only disease was allowed. There was no limit to the number of prior lines of chemotherapy or endocrine therapy in the metastatic setting except for prior treatment with exemestane, which was not allowed.

This study (NCT01594216) was approved by the Institutional Review Board of the University of Pennsylvania. All study procedures were conducted according to the institution’s code of ethics. Written informed consent was obtained from all individual participants in the study.

### Trial design and treatment regimen

This was a prospective phase 2 clinical trial using a Simon 2-stage design^31^. Participants were treated with the combination of ruxolitinib and exemestane on a continuous schedule; a cycle consisted of 28 days duration. All patients received exemestane 25 mg daily. Dosing of ruxolitinib was initially set at 25 mg orally twice daily in the first stage.

### Assessment of tolerability and response

The primary objectives of this trial were to determine the safety and efficacy of the combination of ruxolitinib and exemestane in relapsed, ER+ metastatic BC, using a 2-stage design. Toxicity was evaluated according to the Common Terminology Criteria for Adverse Events (CTCAE) version 4.0.

Tumor response was analyzed utilizing RECIST 1.1. Clinical and radiographic response assessments occurred after every third cycle. Participants with bone-only disease were evaluated for progression by CT or MRI where progression was defined as unequivocal worsening of existing bone lesions and/or appearance of new skeletal or extra-skeletal lesions. Responses were classified as complete response (CR), partial response (PR), or stable disease ≥6 months (SD), and clinical benefit rate (CBR) was defined as the sum of the proportion of patients with CR, PR and SD ≥ 6 months.

Secondary objectives were progression-free survival (PFS) and correlative analyses that included differential response to therapy by measures of the host inflammatory response and estrogen metabolites as well as pharmacologic target inhibition.

### Pharmacodynamic measurement of pSTAT3 inhibition

Phosphorylation of STAT3 and STAT5 in T cells and granulocytes were measured on whole blood from 17 participants who had a baseline and at least one on-treatment trough sample. To measure dynamic range of the assay, 100 μL of whole blood was exposed to varying concentrations of INCB018424 (Ruxolitinib) for 15 minutes at 37 °C (or no ex vivo inhibitor for on treatment samples), and then stimulated for 20 minutes at 37 °C with IL-6 (20 ng/ml), G-CSF (100 ng/ml), or GM-CSF (10–100 ng/ml). After stimulation, the samples were fixed with 4% formaldehyde for 10 minutes at room temperature and permeabilized with 0.1% Triton X-100 for 15 minutes at 37 °C^32^. Next, samples were washed twice in cold PBS supplemented with 4% bovine serum albumin (BSA), treated with cold 100% methanol to enhance epitope availability, and stored at −20 °C. Before analysis, samples were washed twice in cold PBS supplemented with 4% BSA and then incubated with directly labeled antibodies at room temperature for 30 minutes in the dark. Data were acquired on a BD FACSCalibur using CellQuest Pro software and analyzed using FlowJo version 9.3.1. The degree of inhibition of samples from patients on treatment were evaluated by comparing intensity of phosphoSTAT3 and phosphoSTAT5 to pre-treatment samples in the presence of cytokines. To determine the dynamic range of the assay and potential maximal inhibition, a subset (n = 6) of baseline samples were treated with exogenous addition of Ruxolitinib (INCB018424; 5 uM and 10 uM). The best response in treated patients was not significantly different than exogenous addition of 5 uM Ruxolitinib but was significantly less than 10 uM.

#### Measurement of inflammatory markers

Serum concentrations of CRP, IL-6, and SAA were measured at three time points (pretreatment cycle 1 day 1, cycle 1 day 15, and cycle 2 day 1) using the commercially available Luminex quantitative multiplex bead assay (R&D Systems, Minneapolis, MN) and was performed by the Human Immunology Core in the Perelman School of Medicine at the University of Pennsylvania. Analysis of individual serum samples were performed in tandem pairs and back-calculated against a standardized curve repeated on each plate analyzed^7^.

### IL-6 promoter genotyping

IL-6 promoter genotyping methods were adopted from previously described techniques^7^. DNA was extracted from buffy coat samples drawn at the time of enrollment and underwent Sanger sequencing for three functional variants of the IL-6 promoter: −572 G > C (rs1800796), −597 G > A (rs1800797), and −174 G > C (rs1800795). The forward primer sequence was 5’ AAA AAG GAG TCA CAC ACT CCA CCT 3’ and the reverse primer sequence was 5’ TTG GGC TGA TTG GAA ACC TTA TTA 3’. The enzyme used for the PCR reactions was the Roche Expand High Fidelity PCR system (Cat. No 11 759 078 001) and the cycling conditions were as follows: 95 C 5 min; 30 cycles of 95 C 15 s, 57 C 30 s, and 72 C 30 s; 72 C 5 min; then held at 4 C. PCR products were purified with ExoSap. Sequencing reactions were then assembled using BigDye 3.1 (ThermoScientific) and sequenced on ABI 3730XL sequencer in both forward and reverse directions. Based upon prior work assessing the functional impact of these SNPs on IL-6 production^8^, patients were classified as having a “high IL-6” polymorphism if they had G/G genotype in either rs1800795 or rs1800797.

### Measurement of serum estrogen levels

Methods for serum estrogen quantification have been reported elsewhere^7,33^ and again described here. Estrone and estradiol standards were purchased from Steraloids Inc. (Newport, RI). [13C6]-estrone and [13C6]-estradiol were purchased from Cambridge Isotope Laboratories (Cambridge, MA). [13C3]-exemestane was purchased from Isosciences (Ambler, PA) and [2H3]-17β-hydroxy-exemestane was purchased from Toronto Research Chemicals (Toronto, Ontario). β-glucuronidase/arylsulfatase (Helix pomatia) was obtained from Roche (Indianapolis, IN). Dry acetonitrile was purchased from Acros Organic (New Jersey, USA). Methyl-tert-butyl-ether (MTBE), 2-fluoro-1-methylpyridinium p-toluenesulfonate (FMP-TS), triethylamine, methanol, acetone, L-ascorbic acid, formic acid, hydrochloric acid (HCl), sodium chloride, sodium acetate and sodium bicarbonate were obtained from Sigma–Aldrich (Milwaukee, WI)39. Off-the-clot double charcoal-stripped human serum was purchased from Golden West Biologicals, Inc. (Temecula, CA). All solvents were HPLC Optima grade unless otherwise noted.

Off the clot double charcoal-stripped human serum was used as an analytical matrix for the quantification of estrogen metabolites from human serum. An internal standard mix containing [13C6]-estrone, [13C6]-estradiol, [13C3]-exemestane, [2H3]-17β-hydroxy-exemestane was spiked into serum prior to extraction. Calibration curves of estrogens were prepared from standard solutions in the range of 1.56–800 pg/mL. For determination of total estrogens, 10 µL of internal standard working solution was spiked into a 0.1 mL aliquot of serum, followed by the addition of 0.1 mL water, 0.1 mL 0.5% L-ascorbic acid, 0.2 mL sodium acetate buffer (200 mM, pH 5.0), and 20 µL of β-glucuronidase/arylsulfatase. Samples were incubated at 37 °C for 19 h. After hydrolysis, samples were acidified with 15 µL of 1 N HCl followed by addition of 150 µL saturated sodium chloride. Samples underwent liquid–liquid extraction (LLE) with 2.5 mL of MTBE by vortex-mixing for 20 min, followed by centrifugation at 3400 × *g* at 4 °C for 15 min. The upper, organic layer containing extracted estrogens was removed and dried under nitrogen prior to chemical derivatization and LC-HRMS analysis. Formation of methylpyridinium ether derivatives of estrone and estradiol proceeded as follows. 2-fluoro-1-methylpyridinium p-toluenesulfonate (FMP-TS) reagent was freshly prepared at 5 mg/mL in acetonitrile containing 1% triethylamine. Fifty microliter was added to each vial containing extracted estrogens. The mixture was vortexed for 10 s and then incubated at 45 °C for 15 min. The reaction was stopped by the addition of 50 µL water containing 0.1% formic acid and 5 µL of this mixture was directly injected for LC-HRMS analysis.

Separations were performed on a Waters BEH C18 Column (2.1 mm × 50 mm 1.7 μm) using a 7 min gradient starting at 65% methanol w/ 0.1% formic acid. Mobile phase A was water with 0.1% formic acid, and mobile phase B was methanol with 0.1% formic acid. A Thermo QExactive HF instrument was operated in positive ion mode alternating full scan and MS/MS modes at 120,000 resolution. The MS was coupled to an Ultimate 3000 UHPLC interfaced with a heated electrospray ionization (HESI-II) source. Molecular (M+) precursor ions of estrogens were as follows: estrone: 362.2115; [13C6]-estrone: 368.2361; estradiol: 364.2271; and [13C6]-estradiol: 370.2465. The method used the separation of signal from noise based on the molecular ion’s unique stability, by applying extra CID on the parent ion. Serum concentrations of estrogens were calculated using Xcalibur software (version 3.0) from Thermo Fisher Scientific. The limit of detection was 1.0 pg/mL for both E1 and E2. Laboratory staff performing the analyses were blinded to case/control status.

Serum estrogens were reported in both qualitative (estrogens detected or not) and quantitative fashions. Subjects with serum E1 or E2 levels that were below the limit of detection were reported as 0.2 pg/mL (or the limit of detection/5) so they could be included in the quantitative analysis.

### Statistical analysis

Given the fact that there were no prior studies of this combination, the Simon 2-stage design^31^ was employed to evaluate toxicity and determine sample size as follows: if five or more participants in the first 15 enrolled and evaluable experienced grade 3 or 4 toxicity requiring discontinuation from the study in the first treatment cycle (defined as 28 days duration) the trial would not proceed to the second stage. If fewer than five participants experienced grade 3 or 4 toxicity requiring discontinuation, an additional 10 participants would be enrolled in the second stage, with the dose of ruxolitinib modified as needed based on the initial toxicity evaluation.

Precision estimates for responses were calculated. With 25 participants and a pre-set alpha level of 0.027, the trial had 80% power to detect a response rate of 25%. For the correlative analyses, the primary dichotomous response variable was defined as stable disease at 6 months (yes/no). Inflammatory serum markers (continuous: CRP, IL-6, SAA), baseline estrone/estradiol levels (continuous), and IL-6 promoter genotype (categorical: high-risk vs low-risk) were the predictor variables. Predictor variables were tested for normality using the Shapiro-Wilk test for normality. As the continuous predictor variables did not follow a normal distribution, non-parametric testing was employed to test for significant differences in levels of inflammatory serum markers by response group. Effect sizes and confidence intervals were calculated for each predictor variable using univariate logistic regression. All tests for significance were two-sided with an alpha level of 0.05. Analyses were carried out in STATA IC/16.1.

### Reporting summary

Further information on research design is available in the Nature Research Reporting Summary linked to this article.

## Supplementary information

Supplementary Figures

Reporting Summary

## Data Availability

The source data that informed the analysis, results, and figures in this manuscript are openly available as part of the following data record: https://figshare.com/projects/Makhlin_et_al_JAKEE_source_data/139486.

## References

[1] Baum M (2002). Anastrozole alone or in combination with tamoxifen versus tamoxifen alone for adjuvant treatment of postmenopausal women with early breast cancer: First results of the ATAC randomised trial. Lancet.

[2] Matikas A, Foukakis T, Bergh J (2017). Tackling endocrine resistance in ER-positive HER2-negative advanced breast cancer: A tale of imprecision medicine. Crit. Rev. Oncol./Hematol..

[3] Bromberg J (2000). Signal transducers and activators of transcription as regulators of growth, apoptosis and breast development. Breast Cancer Res..

[4] Bromberg J (2002). Stat proteins and oncogenesis. J. Clin. Invest..

[5] Pierce BL (2009). Elevated biomarkers of inflammation are associated with reduced survival among breast cancer patients. J. Clin. Oncol..

[6] Villaseñor A (2014). Postdiagnosis c-reactive protein and breast cancer survivorship: Findings from the WHEL study. Cancer Epidemiol. Biomark. Prev..

[7] McAndrew, N. P. et al. Effects of systemic inflammation on relapse in early breast cancer. *npj Breast Cancer* **7**, 7 (2021). 10.1038/s41523-020-00212-6 PMC7822844 33483516

[8] DeMichele A (2009). Host genetic variants in the interleukin-6 promoter predict poor outcome in patients with estrogen receptor-positive, node-positive breast cancer. Cancer Res..

[9] Dethlefsen C, Højfeldt G, Hojman P (2013). The role of intratumoral and systemic IL-6 in breast cancer. Breast Cancer Res. Treat..

[10] Ham M, Moon A (2013). Inflammatory and microenvironmental factors involved in breast cancer progression. Arch. Pharmacal Res..

[11] Siersbæk R (2020). IL6/STAT3 Signaling Hijacks Estrogen Receptor α Enhancers to Drive Breast Cancer Metastasis. Cancer Cell.

[12] Sansone, P. et al. Self-renewal of CD133(hi) cells by IL6/Notch3 signalling regulates endocrine resistance in metastatic breast cancer. *Nat. Commun*. **7**, 10442 (2016). 10.1038/ncomms10442 PMC4748123 26858125

[13] Bose P, Verstovsek S (2017). JAK2 inhibitors for myeloproliferative neoplasms: What is next?. Blood.

[14] Stover DG (2018). Phase II study of ruxolitinib, a selective JAK1/2 inhibitor, in patients with metastatic triple-negative breast cancer. npj Breast Cancer.

[15] O’Shaughnessy J (2018). A randomized, double-blind, phase 2 study of ruxolitinib or placebo in combination with capecitabine in patients with advanced HER2-negative breast cancer and elevated C-reactive protein, a marker of systemic inflammation. Breast Cancer Res. Treat..

[16] Lynce F (2021). Phase I study of JAK1/2 inhibitor ruxolitinib with weekly paclitaxel for the treatment of HER2-negative metastatic breast cancer. Cancer Chemother. Pharmacol..

[17] Kearney M (2021). Phase I/II trial of ruxolitinib in combination with trastuzumab in metastatic HER2 positive breast cancer. Breast Cancer Res. Treat..

[18] Buzdar AU, Robertson JFR, Eiermann W, Nabholtz JM (2002). An overview of the pharmacology and pharmacokinetics of the newer generation aromatase inhibitors anastrozole, letrozole, and exemestane. Cancer.

[19] Jones SA, Jones SE (2000). Exemestane: a novel aromatase inactivator for breast cancer. Clin. breast cancer.

[20] Lonning PE (2000). Pharmacology and clinical experience with exemestane. Expert Opin. Investig. Drugs.

[21] Lønning PE (2001). Exemestane: A review of its clinical efficacy and safety. Breast.

[22] Chia S (2008). Double-blind, randomized placebo controlled trial of fulvestrant compared with exemestane after prior nonsteroidal aromatase inhibitor therapy in postmenopausal women with hormone receptor-positive, advanced breast cancer: Rsults from EFECT. J. Clin. Oncol..

[23] Kalota, A., Jeschke, G. R., Carroll, M. & Hexner, E. O. Intrinsic Resistance to JAK2 Inhibition in Myelofibrosis. *Clin. Cancer Res.* **19**, 1729–1739 (2013). 10.1158/1078-0432.CCR-12-1907 PMC3618591 23386690

[24] Bidard F-C (2019). Emergence of ESR1 mutation in cell-free DNA during first line aromatase inhibitor and palbociclib: An exploratory analysis of the PADA-1 trial. Ann. Oncol..

[25] Stoenescu, A. et al. Serum C-reactive protein as potential independent prognostic factor for breast cancer. *Italian J. Gynaecol. Obstetrics* **27**, 1151–1159 (2015).

[26] Hartman ZC (2011). HER2 overexpression elicits a proinflammatory IL-6 autocrine signaling loop that is critical for tumorigenesis. Cancer Res..

[27] Singh, A. et al. Control of Aromatase Activity in Breast Tumours: the Role of the Immune System. *J. Steroid Biochem*. *Molec. Biol.* **61**, 185–192 (1997). 9365189

[28] Purohit A (1995). Aromatase activity and interleukin-6 production by normal and malignant breast tissues. J. Clin. Endocrinol. Metab..

[29] Woo P, Humphries SE (2013). IL-6 polymorphisms: A useful genetic tool for inflammation research?. J. Clin. Invest..

[30] Lorente, L. et al. Association between Interleukin-6 Promoter Polymorphism (−174 G/C), Serum Interleukin-6 Levels and Mortality in Severe Septic Patients. *Int. J. Mol. Sci*. **17**, 1861 (2016). 10.3390/ijms17111861 PMC5133861 27834822

[31] Simon R (1989). Optimal two-stage designs for phase II clinical trials. Control. Clin. Trials.

[32] Chow S, Minden MD, Hedley DW (2006). Constitutive phosphorylation of the S6 ribosomal protein via mTOR and ERK signaling in the peripheral blasts of acute leukemia patients. Exp. Hematol..

[33] AMM F (2016). Derivatization of estrogens enhances specificity and sensitivity of analysis of human plasma and serum by liquid chromatography tandem mass spectrometry. Talanta.

