# Supplementary material for: Ruxolitinib and exemestane for estrogen receptor positive, aromatase inhibitor resistant advanced breast cancer
Source: NPJ Breast Cancer. 2022 Nov 11;8:122. doi: 10.1038/s41523-022-00487-x (PMC9652412; doi:10.1038/s41523-022-00487-x)
Supplement: Supplementary file 1 — Supplementary Figures [file 41523_2022_487_MOESM1_ESM.pdf]

Supplementary Figures

Supplementary Figure 1

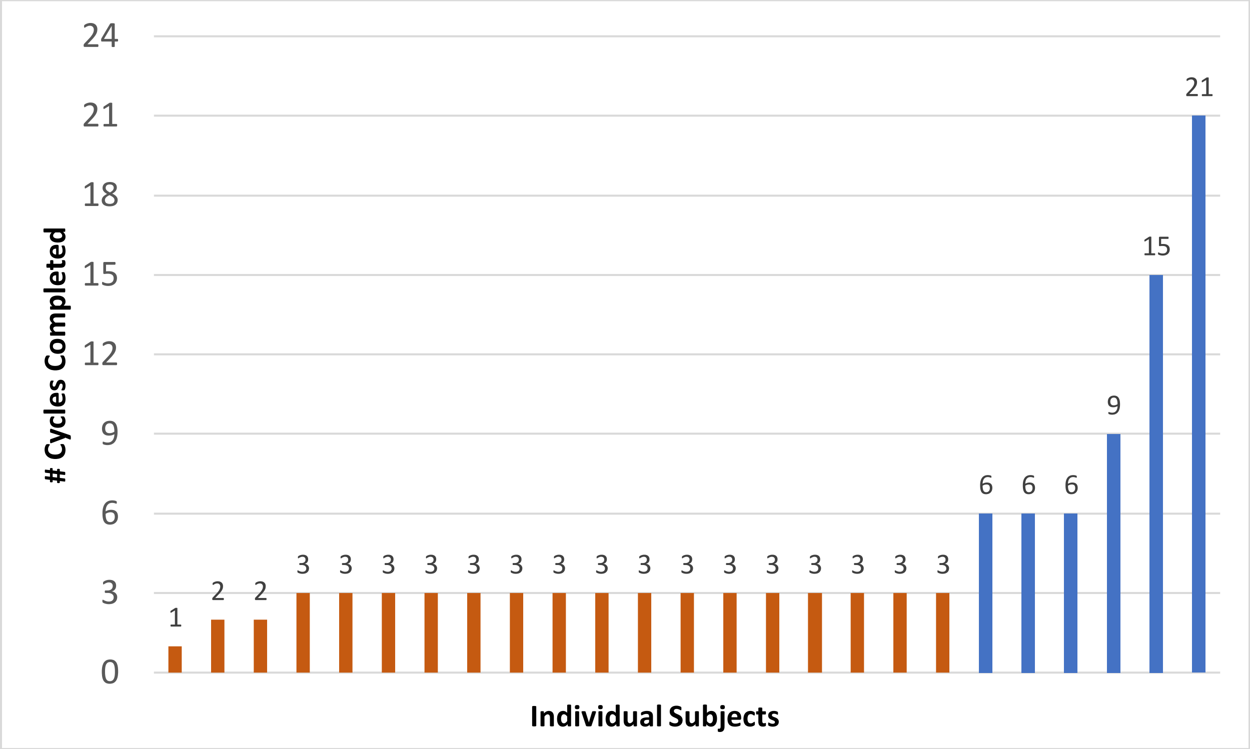

Number of Cycles Completed by Subject *Plot demonstrating number of cycles completed on study for each participant. Responses here are grouped into progression prior to cycle 6 (Orange) and stable disease ≥6 cycles (blue).*

Supplementary Figure 2

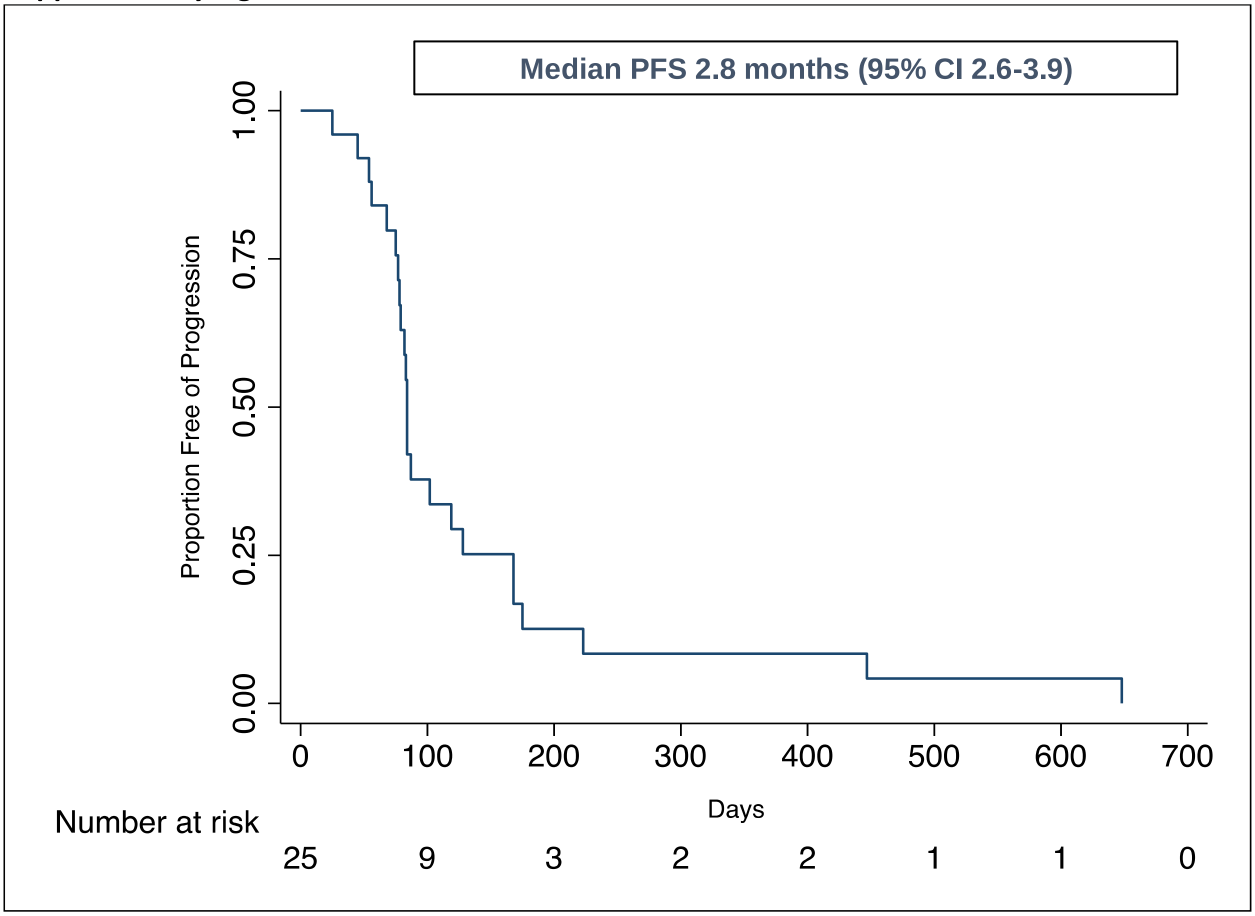

Progression-free Survival Kaplan Meier Plot *Kaplan Meier Plot showing progression-free survival.*

Supplementary Figure 3

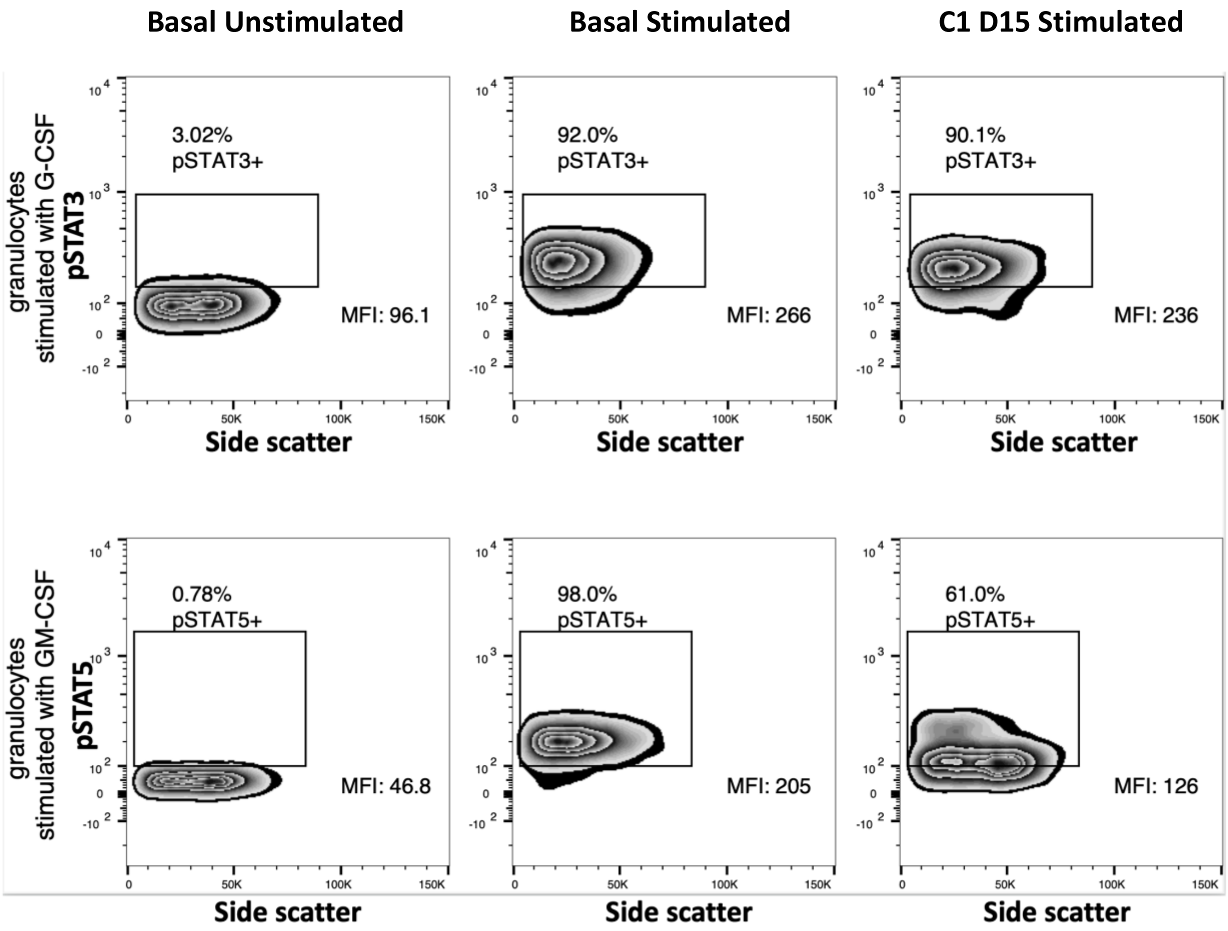

Pharmacodynamic Analysis of Ruxolitinib Target Inhibition *In granulocytes stimulated with G-CSF, all samples showed some attenuation of phosphoSTAT3 in treated samples (top right panel, median inhibition: 31%, range 12-90%). 13 of 17 samples had a blunting of phosphoSTAT5 in response to GM-CSF (bottom right panel, median inhibition: 21 %, range 0-65%). MFI = Mean Fluorescent Intensity.*

Supplementary Figure 4

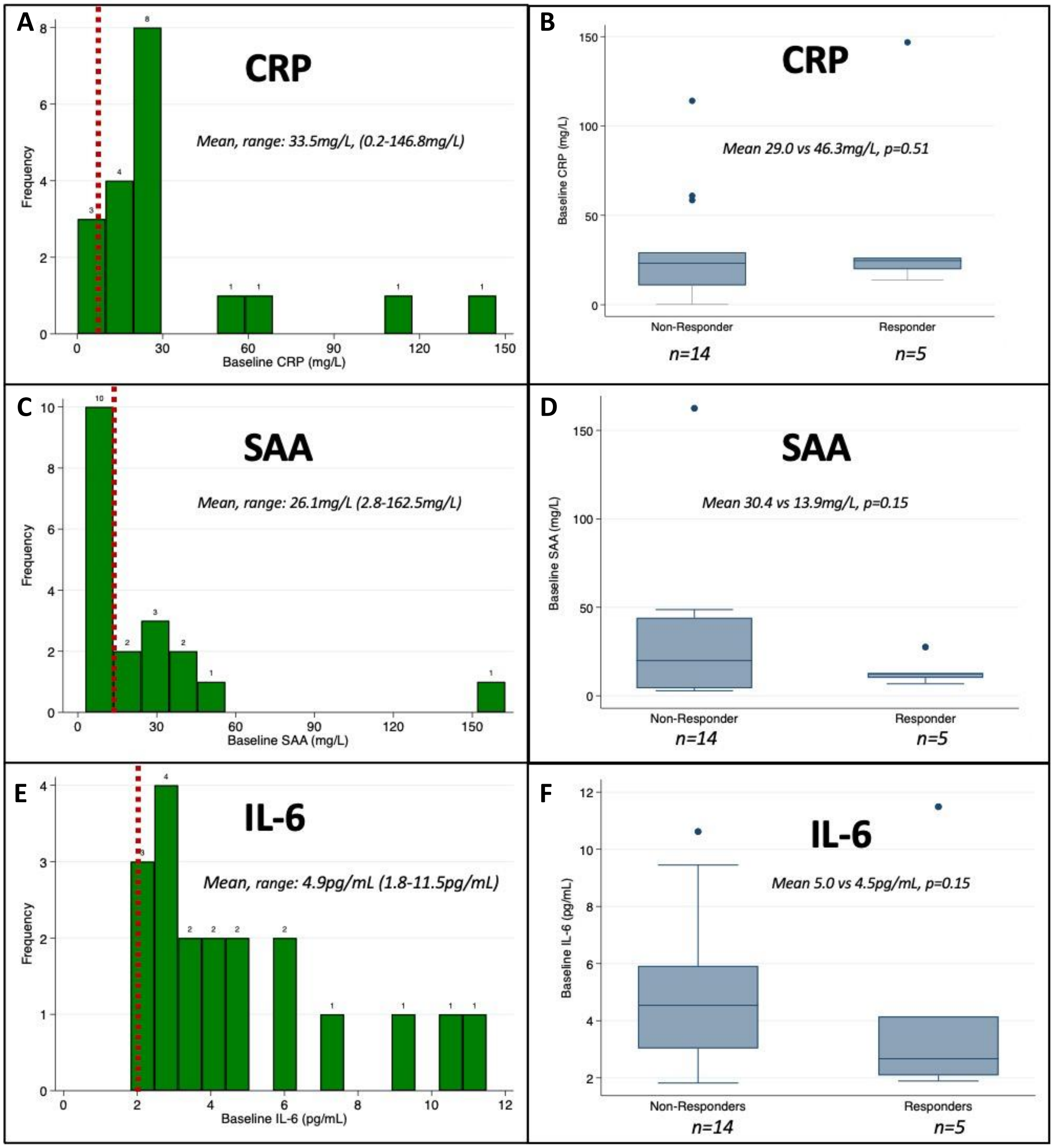

Inflammatory Marker Histograms *Left panels (A, C, E) shows histograms for baseline CRP, SAA, and IL-6 levels. Dashed red line indicates the upper limit of normal defined by the laboratory medicine at the Hospital of the University of Pennsylvania. The baseline levels for all 3 biomarkers were elevated. Frequencies above each bar represent number of participants. Right panels (B,D,F) show box plot distributions of baseline CRP, SAA and IL-6 by responder groups. Whisker endpoints represent range (minimum-maximum), box limits represent interquartile range (upper and lower quartiles), and center line represents the median. Points above the box-and-whiskers represent outliers. P-values represent non-parametric equality of median test performed to compare medians of baseline values of CRP, SAA, and IL-6 between responder groups.*

Supplementary Figure 5

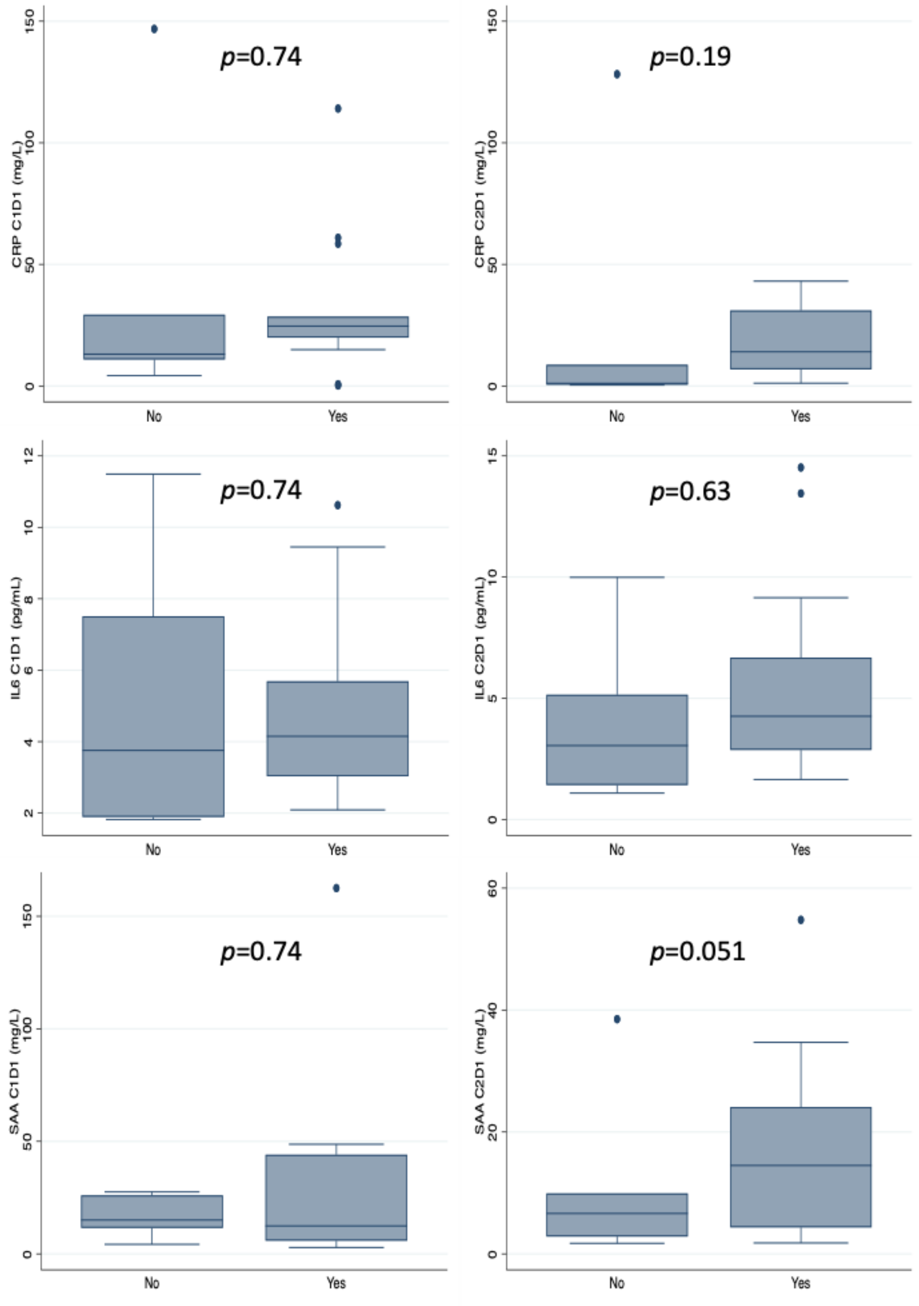

Inflammatory Markers by IL-6 Promoter Status *Box plots showing baseline inflammatory markers (CRP, IL-6, SAA) by IL-6 promoter status (high-risk or not). Whisker endpoints represent range (minimum-maximum), box limits represent interquartile range (upper and lower quartiles), and center line represents the median. Points above the whiskers and boxes represent outliers. P-values represent non-parametric equality of median test.*

Supplementary Figure 6

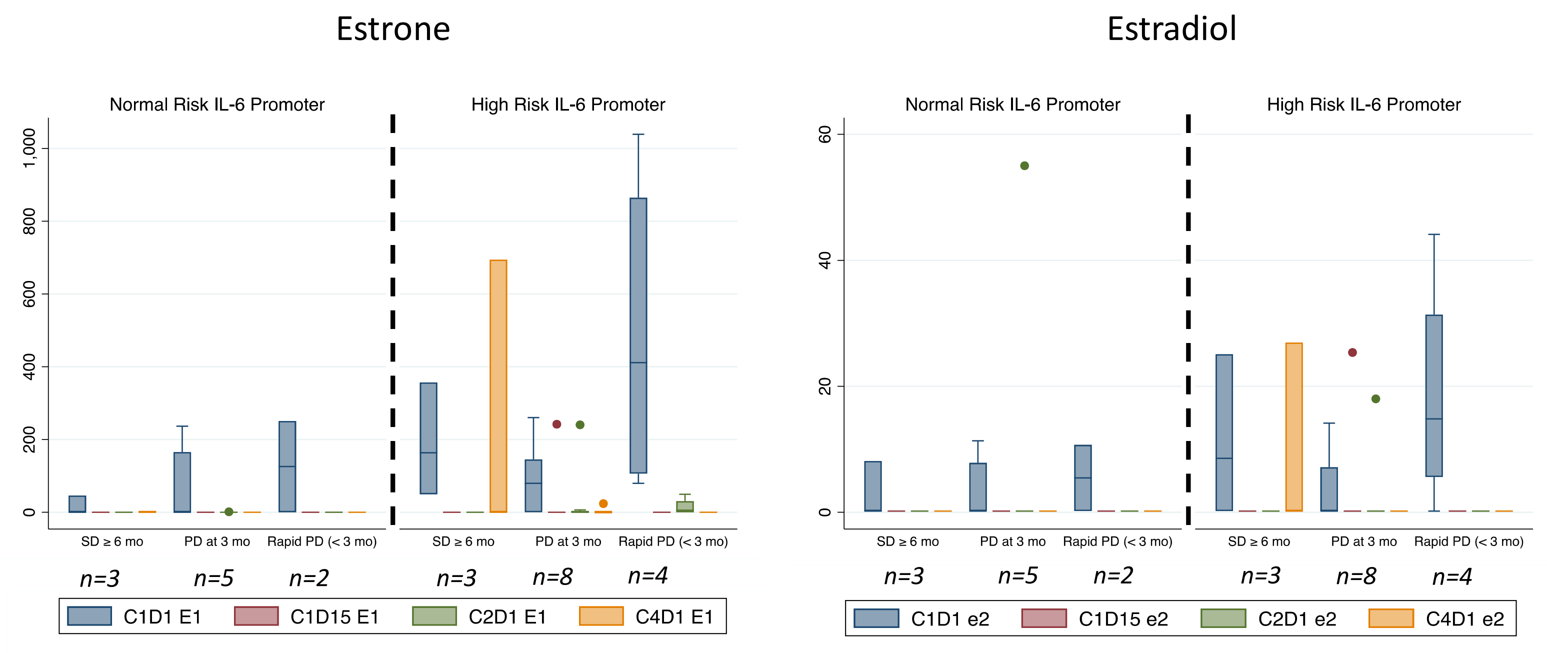

Estrogen Levels by IL-6 Promoter Status and Response Group *Box plots for estrone and estradiol levels, stratified by IL-6 promoter status and by responder groups (stable disease ≥6 months, progressive disease at 3 months, and rapid progression). Estrone and estradiol levels measured at baseline (C1D1), cycle 1 day 15 (C1D15), cycle 2 day 1 (C2D1), and cycle 4 day 1 (C4D1). Whisker endpoints represent range (minimum-maximum), box limits represent interquartile range (upper and lower quartiles), and center line represents the median. Points above the whiskers and boxes represent outliers.*
