# Supplementary material for: Ruxolitinib and exemestane for estrogen receptor positive, aromatase inhibitor resistant advanced breast cancer
Source: NPJ Breast Cancer. 2022 Nov 11;8:122. doi: 10.1038/s41523-022-00487-x (PMC9652412; doi:10.1038/s41523-022-00487-x)
Supplement: Supplementary file 2 — Reporting Summary [file 41523_2022_487_MOESM2_ESM.pdf]

nature research

Corresponding author(s): Igor Makhlin, MD

Last updated by author(s): Aug 1, 2022

Reporting Summary

Nature Research wishes to improve the reproducibility of the work that we publish. This form provides structure for consistency and transparency in reporting. For further information on Nature Research policies, see our Editorial Policies and the Editorial Policy Checklist.

Statistics

For all statistical analyses, confirm that the following items are present in the figure legend, table legend, main text, or Methods section.

n/a	Confirmed	
☐	☒	The exact sample size (n) for each experimental group/condition, given as a discrete number and unit of measurement
☒	☐	A statement on whether measurements were taken from distinct samples or whether the same sample was measured repeatedly
☐	☒	The statistical test(s) used AND whether they are one- or two-sided<br>*Only common tests should be described solely by name; describe more complex techniques in the Methods section.*
☐	☒	A description of all covariates tested
☐	☒	A description of any assumptions or corrections, such as tests of normality and adjustment for multiple comparisons
☐	☒	A full description of the statistical parameters including central tendency (e.g. means) or other basic estimates (e.g. regression coefficient) AND variation (e.g. standard deviation) or associated estimates of uncertainty (e.g. confidence intervals)
☒	☐	For null hypothesis testing, the test statistic (e.g. F, t, r) with confidence intervals, effect sizes, degrees of freedom and P value noted<br>*Give P values as exact values whenever suitable.*
☒	☐	For Bayesian analysis, information on the choice of priors and Markov chain Monte Carlo settings
☒	☐	For hierarchical and complex designs, identification of the appropriate level for tests and full reporting of outcomes
☒	☐	Estimates of effect sizes (e.g. Cohen's d, Pearson's r), indicating how they were calculated

Our web collection on statistics for biologists contains articles on many of the points above.

Software and code

Policy information about availability of computer code

Data collection	N/A
Data analysis	Analyses were carried out in STATA IC/16.1.

For manuscripts utilizing custom algorithms or software that are central to the research but not yet described in published literature, software must be made available to editors and reviewers. We strongly encourage code deposition in a community repository (e.g. GitHub). See the Nature Research guidelines for submitting code & software for further information.

Data

Policy information about availability of data

All manuscripts must include a data availability statement. This statement should provide the following information, where applicable:

- Accession codes, unique identifiers, or web links for publicly available datasets
- A list of figures that have associated raw data
- A description of any restrictions on data availability

The source data that informed the analysis, results, and figures in this manuscript are openly available as part of the following data record: https://figshare.com/projects/Makhlin_et_al_JAKEE_source_data/139486

Field-specific reporting

Please select the one below that is the best fit for your research. If you are not sure, read the appropriate sections before making your selection.

☒ Life sciences ☐ Behavioural & social sciences ☐ Ecological, evolutionary & environmental sciences

For a reference copy of the document with all sections, see nature.com/documents/nr-reporting-summary-flat.pdf

Life sciences study design

All studies must disclose on these points even when the disclosure is negative.

Sample size	Given the fact that there were no prior studies of this combination, the 2-stage design was employed to evaluate toxicity as follows: if five or more participants in the first 15 enrolled and evaluable experienced grade 3 or 4 toxicity requiring discontinuation from the study in the first treatment cycle (defined as 28 days duration) the trial would not proceed to the second stage. If fewer than five participants experienced grade 3 or 4 toxicity requiring discontinuation, an additional 10 participants would be enrolled in the second stage, with the dose of ruxolitinib modified as needed based on the initial toxicity evaluation. Precision estimates for responses were calculated. With 25 participants and a pre-set alpha level of 0.027, the trial had 80% power to detect a response rate of 25%
Data exclusions	No data were excluded from this analysis.
Replication	This was a phase 2 single arm clinical trial, which did not meet the efficacy endpoint for further study. Therefore, we do not expect that this clinical trial will be replicated or repeated with larger numbers given our pharmacodynamic studies demonstrating only modest on-target effect of the investigational agent ruxolitinib on the IL-6/STAT3 pathway. We utilized a straight-forward and common 2-stage design which has been reproduced in countless early phase clinical trials.
Randomization	This was a single arm clinical trial. There was no randomized component.
Blinding	This was a single arm clinical trial. Therefore, neither investigators nor participants were blinded to the investigational combination of exemestane + ruxolitinib.

Reporting for specific materials, systems and methods

We require information from authors about some types of materials, experimental systems and methods used in many studies. Here, indicate whether each material, system or method listed is relevant to your study. If you are not sure if a list item applies to your research, read the appropriate section before selecting a response.

Materials & experimental systems

n/a	Involved in the study	
☒	☐	Antibodies
☒	☐	Eukaryotic cell lines
☒	☐	Palaeontology and archaeology
☒	☐	Animals and other organisms
☐	☒	Human research participants
☐	☒	Clinical data
☒	☐	Dual use research of concern

Methods

n/a	Involved in the study	
☒	☐	ChIP-seq
☐	☒	Flow cytometry
☒	☐	MRI-based neuroimaging

Human research participants

Policy information about studies involving human research participants

Population characteristics	Eligible participants (all female) had histologically confirmed metastatic ER+ breast cancer (defined as ≥5% by immunohistochemistry) on either a primary or metastatic tumor biopsy and were post-menopausal (either surgically via oophorectomy or no menses in the previous 12-month period). Those that were also HER2+ by IHC (3+) or FISH (by ASCO/CAP guidelines) were eligible to participate. Patients who were premenopausal at diagnosis and rendered amenorrheic by tamoxifen were required to have a serum estradiol level <30 pg/ml after discontinuation of tamoxifen. Participants must have either relapsed within 2 years of completing adjuvant non-steroidal aromatase inhibitor or progressed on one in the metastatic setting. Bone-only disease was allowed. There was no limit to the number of prior lines of chemotherapy or endocrine therapy in the metastatic setting with the exception of prior treatment with exemestane, which was not allowed. IL-6 genotype was not known a priori, but was measured on study and 60% of participants harbored a high-risk IL-6 promoter polymorphism.
Recruitment	Participants were recruited at the Hospital of the University of Pennsylvania where they receive breast cancer care, and by referral from oncologists outside of the Penn health system.

Ethics oversight | Institutional Review Board of the University of Pennsylvania

Note that full information on the approval of the study protocol must also be provided in the manuscript.

Clinical data

Policy information about clinical studies

All manuscripts should comply with the ICMJE guidelines for publication of clinical research and a completed CONSORT checklist must be included with all submissions.

Clinical trial registration: NCT01594216

Study protocol: https://figshare.com/account/projects/139486/articles/20444607

Data collection: Participants were recruited at the Hospital of the University of Pennsylvania, Abramson Cancer Center, and enrolled from 10/22/12 to 1/14/16. This was a single institution study single arm trial; data was collected and maintained by the primary research team led by Dr. DeMichele at the University of Pennsylvania.

Outcomes: As this was a single arm trial testing the combination of exemestane and ruxolitinib, which have not been combined previously, the primary objectives of this trial were to determine the safety and efficacy of the combination in relapsed, ER+ metastatic breast cancer using a 2-stage design. Toxicity was evaluated according to the Common Terminology Criteria for Adverse Events (CTCAE) version 4.0. Tumor response was analyzed utilizing RECIST 1.1. Clinical and radiographic response assessments occurred after every third cycle. Participants with bone-only disease were evaluated for progression by CT or MRI where progression was defined as unequivocal worsening of existing bone lesions and/or appearance of new skeletal or extra-skeletal lesions. Responses were classified as complete response (CR), partial response (PR), or stable disease ≥6 months (SD), and clinical benefit rate (CBR) was defined as the sum of the proportion of patients with CR, PR and SD ≥6 months.
Secondary objectives included assessing for differential response to therapy by measures of the host inflammatory response and estrogen metabolites as well as to test pharmacologic target inhibition using a flow cytometry-based assay.

Flow Cytometry

Plots

Confirm that:

- [ ] The axis labels state the marker and fluorochrome used (e.g. CD4-FITC).
- [ ] The axis scales are clearly visible. Include numbers along axes only for bottom left plot of group (a 'group' is an analysis of identical markers).
- [x] All plots are contour plots with outliers or pseudocolor plots.
- [x] A numerical value for number of cells or percentage (with statistics) is provided.

Methodology

Sample preparation: Samples from participants were treated with IL-6, G-CSF and GM-CSF (10-100 ng/ml) to induce cytokine stimulation and downstream STAT3/5 phosphorylation. To measure dynamic range of the assay, 100 µL of whole blood was exposed to varying concentrations of INCB018424 (Ruxolitinib) for 15 minutes at 37°C (or no ex vivo inhibitor for on treatment samples), and then stimulated for 20 minutes at 37°C with IL-6 (20 ng/ml), G-CSF (100 ng/ml), and GM-CSF (10-100 ng/ml). After stimulation, the samples were fixed with 4% formaldehyde for 10 minutes at room temperature and permeabilized with 0.1% Triton X-100 for 15 minutes at 37°C. Next, samples were washed twice in cold PBS supplemented with 4% bovine serum albumin (BSA), treated with cold 100% methanol to enhance epitope availability, and stored at −20°C. Before analysis, samples were washed twice in cold PBS supplemented with 4% BSA and then incubated with directly labeled antibodies at room temperature for 30 minutes in the dark.

Instrument: Data were acquired on a BD FACSCalibur

Software: CellQuest Pro software and analyzed using FlowJo version 9.3.1.

Cell population abundance: *Describe the abundance of the relevant cell populations within post-sort fractions, providing details on the purity of the samples and how it was determined.*

Gating strategy: *Describe the gating strategy used for all relevant experiments, specifying the preliminary FSC/SSC gates of the starting cell population, indicating where boundaries between "positive" and "negative" staining cell populations are defined.*

- [ ] Tick this box to confirm that a figure exemplifying the gating strategy is provided in the Supplementary Information.
